# Therapeutic Target Identification and Inhibitor Screening against Riboflavin Synthase of Colorectal Cancer Associated *Fusobacterium nucleatum*

**DOI:** 10.3390/cancers14246260

**Published:** 2022-12-19

**Authors:** Norah A. Alturki, Mutaib M. Mashraqi, Khurshid Jalal, Kanwal Khan, Zarrin Basharat, Ahmad Alzamami

**Affiliations:** 1 Clinical Laboratory Science Department, College of Applied Medical Sciences, King Saud University, Riyadh 11433, Saudi Arabia; 2 Department of Clinical Laboratory Sciences, College of Applied Medical Sciences, Najran University, Najran 61441, Saudi Arabia; 3 HEJ Research Institute of Chemistry, International Center for Chemical and Biological Sciences, University of Karachi, Karachi 75270, Pakistan; 4 Dr. Panjwani Center for Molecular Medicine and Drug Research, International Center for Chemical and Biological Sciences, University of Karachi, Karachi 75270, Pakistan; 5 Jamil-ur-Rahman Center for Genome Research, Dr. Panjwani Center for Molecular Medicine and Drug Research, International Center for Chemical and Biological Sciences, University of Karachi, Karachi 75270, Pakistan; 6 Clinical Laboratory Science Department, College of Applied Medical Sciences, Shaqra University, Al-Quwayiyah 11961, Saudi Arabia

**Keywords:** pan-genome, *F. nucleatum*, colorectal cancer, riboflavin synthase, virtual screening, computer aided drug design, natural products

## Abstract

**Simple Summary:**

More and more studies are suggesting the role of microbes in several diseases in addition to the germline and environmental factors. *F. nucleatum* is recently being associated with colorectal cancer and here, we aimed to identify important drug targets from the core genome of colorectal cancer associated *F. nucleatum* through bioinformatics approach. We used one drug target for further analysis and obtained natural product inhibitors against it. Finally, we validated inhibition stability through dynamics simulation approach. We are hopeful that this study could benefit researchers working on colorectal cancer, its microbiome and cure.

**Abstract:**

Colorectal cancer (CRC) ranks third among all cancers in terms of prevalence. There is growing evidence that gut microbiota has a role in the development of colorectal cancer. *Fusobacterium nucleatum* is overrepresented in the gastrointestinal tract and tumor microenvironment of patients with CRC. This suggests the role of *F. nucleatum* as a potential risk factor in the development of CRC. Hence, we aimed to explore whole genomes of *F. nucleatum* strains related to CRC to predict potential therapeutic markers through a pan-genome integrated subtractive genomics approach. In the current study, we identified 538 proteins as essential for *F. nucleatum* survival, 209 non-homologous to a human host, and 12 as drug targets. Eventually, riboflavin synthase (RiS) was selected as a therapeutic target for further processing. Three different inhibitor libraries of lead-like natural products, i.e., cyanobactins (*n* = 237), streptomycins (*n* = 607), and marine bacterial secondary metabolites (*n* = 1226) were screened against it. After the structure-based study, three compounds, i.e., CMNPD3609 (−7.63) > Malyngamide V (−7.03) > ZINC06804365 (−7.01) were prioritized as potential inhibitors of *F. nucleatum*. Additionally, the stability and flexibility of these compounds bound to RiS were determined via a molecular dynamics simulation of 50 ns. Results revealed the stability of these compounds within the binding pocket, after 5 ns. ADMET profiling showed compounds as drug-like, non-permeable to the blood brain barrier, non-toxic, and HIA permeable. Pan-genomics mediated drug target identification and the virtual screening of inhibitors is the preliminary step towards inhibition of this pathogenic oncobacterium and we suggest mouse model experiments to validate our findings.

## 1. Introduction

Colorectal cancer (CRC) is the world’s third most common malignant neoplasm and the fourth leading cause of cancer mortality, with a five-year survival rate of about 65% [1]. Statistically, the mortality rate of CRC has decreased in places with high level of healthcare resources, while it has increased in areas with low levels of medical resources. Several variables contribute to the development of colorectal cancer, including hereditary and environmental factors such as diet and lifestyle [2,3,4]. The progression from precancerous adenomatous polyps to adenocarcinoma generally takes about 10 years [5], and is considered to be the consequence of host mutations that accumulate over time. Improved methods for detecting microorganisms and new insights into the human microbiome have led to a new perspective on several diseases, including CRC [6,7,8]. According to recent research, this cancer is linked with altered gut microbiota [9] and aided by the anaerobic gram-negative bacteria *F. nucleatum* [4,10,11,12]. It is often linked to advanced disease, chemoresistance, distant metastases, and a poor prognosis [13,14,15]. Additional studies have demonstrated that *F. nucleatum* promotes carcinogenesis, changes lymphocyte infiltration of tumor-infiltrating lymphocytes, suppresses NK cell and tumor-infiltrating T cell death, and enhances resistance to chemotherapy in colon cancer [7,15,16]. *Fusobacteria* linked to CRC develops in the oral microbiome and reaches the colon through hematogenous rather than gastrointestinal routes. It has been found in approximately 10–90% of CRC tissue samples, with a higher prevalence in the distal colon than the proximal one. Baik et al. [17] have identified IgA antibodies against the amyloid adhesin factor FadA of this bacterium in the colorectal neoplasia. Cavallucci and colleagues [18] reported that *F. nucleatum* binds Gal-GalNac disaccharide and CECAM-1 on the CRC tumor stem cells, leading to the activation of a MAP kinase. MAP kinase has an implied role in the CRC, therefore, this gives *F. nucleatum* a strong linkage with CRC progression [19,20]. Gao et al. [21] have shown that *F. nucleatum* asserts a positive impact on PD-L1 (an immune frontier point) by modifying an oncogene IFIT1. This causes immunosuppression and tumor progression in the CRC [21]. Lacourse et al. [22] have also shown that the chemotherapeutic agent 5-fluorouracil is an inhibitor of this oncomicrobe but if *E. coli* modifies this compound, then the growth of tumor and this bacterium remains upward. This demonstrates the importance of targeting bacteria alongside the tumor in CRC. Chen et al. [23] have explored CRC therapy options by targeting microbiota, particularly *F. nucleatum*, as infected tumors have shown a significant upsurge in the nitroreductase activity of this bacteria [23]. They instructed an enzyme assembly to dissolve and dismantle tumors through this approach in a mouse model and observed successful results.

In Saudi Arabia (SA), the age-standardized incidence rates (ASIR) and age-standardization mortality rate (ASMR) for all cancers are 88.7 and 43.3, respectively, per 100,000 people. After breast cancer (ASIR-27.3, ASMR-7.5), colorectal cancer (ASIR-13.1, ASMR-6.3) is the second most frequent cancer in Saudi Arabia. According to the GLOBOCON 2018 report, the highest ASMR and ASIR were discovered in Qatar and Oman, respectively, while the greatest ASIR was recorded in the UAE, Saudi Arabia, and Kuwait, according to ICAR, WHO, and GLOBOCON 2018 [14,24,25]. To cope with this disease burden, new therapeutic biomarkers and drug targets are required. New targets may have useful mechanisms [26] that can be harnessed to render the bacteria inactive when bound by drug. Next-generation sequencing (NGS) technology has made it simpler to acquire genetic data of microbial species [8]. Previously, only a small number of genome sequences were available, but now the complete genetic repertoire of organisms may be explored [8,27]. One of the best strategies in the drug development pipeline is to identify therapeutic targets in pathogens using a bioinformatics approach. This is a swift method. Aside from being expensive and time-consuming, conventional drug development in wet laboratories suffers from other drawbacks. An organism’s whole genetic repertoire cannot be discerned by a single genomic sequence, therefore the advancement of big data and data mining-based informatics approaches has a huge advantage [25,28,29]. Pan-genomic studies, which account for the whole set of genes across all strains within a genus, have become possible because of recent algorithmic and computational power developments [30]. Understanding the evolution of a strain requires knowledge of its whole genome sequence, and information of conserved fractions will also enable more specific targets for therapy and inhibitor development. Recently, this has been implemented against gram-negative bacteria such as *Brachypodium hybridum* [31], *Klebsiella pneumoniae* [32], and *Shigella* sp. [28,33]. Subtractive genome analysis is an effective method of finding the gene or group of genes responsible for a certain characteristic in an organism [34]. It helps discover the systemic variety in a given organism by analyzing its full genomic array.

Therefore, to address the challenge of processing data from the wide range of *F. nucleatum* genomes associated with CRC, we did a pan-genomic analysis and examined a large number of *F. nucleatum* pathovar isolates linked with CRC (*n* = 14) to characterize the core and accessory-genome subsets and prioritize therapeutic targets. To our knowledge, this is the first pan-genome mediated study on numerous *F. nucleatum* species linked with CRC, as well as the use of core gene data to investigate therapeutic targets in them. Furthermore, three different libraries of natural products were used to identify potent inhibitors that may be helpful to combat CRC-linked *Fusobacterium* sp.

## 2. Methodology

### 2.1. Data Retrieval

An extensive literature survey was performed to retrieve CRC-related *F. nucleatum* genomes for pan-genome analysis. Fourteen *F. nucleatum* genomes were used in this study (Table 1), derived either from the colon cancer site, tissue, or gut of the ailing person. Sub-species included *animalis*, *polymorphum*, and *vincentii*. The Universal Protein Resource (UniProt) database was used to download the human proteome. The significance of therapeutic targets was examined using the Database of Essential Genes (DEG) [35] and the Cluster of Essential Genes (CEG) [36]. The most recent 2022–3 edition of the DrugBank database [37] was used to assess the potential for the druggability of the proteins. Additionally, for the structure-based virtual screening, the cyanobactins (*n* = 237), streptomycins (*n* = 607), and marine bacterial secondary metabolites (*n* = 1226) were utilized.

### 2.2. Pan-Genome and Core Genome Analysis

The retrieved 14 CRC-related *F. nucleatum* strains were subjected to pan-genome profiling to analyze the variation in the genome content (core genome and dispensable genome) using the BGPA software version 1.2, with parameters described before [43]. A USEARCH clustering algorithm was utilized to cluster only homologous genes detected in the pan-genome, with a 70% cut-off value. The genomes were aligned using the MUSCLE program [44] with default settings, and the phylogenetic analysis and tree-building processes were carried out using UPGMA. Following the classification of gene homologous sets, functional annotation was carried out using the Cluster of Ortholog Groups (COG) [45].

### 2.3. Potential Drug Targets Identification

Therapeutic targets were discovered by subtractive genomic analysis, using the predicted core genome of *F. nucleatum*. CD-HIT with a sequence identity cutoff of 0.8 (i.e., 80%) was performed to identify paralogous or duplicate protein sequences. The non-paralogous sequences of the *F. nucleatum* genome were removed from the core genome fraction of *F. nucleatum.*

These sequences were then subjected to BLASTP with an E-value cutoff of 10^−4^ [46] against the whole human proteome. The proteins with an E-value less than 10^−3^ were examined as potential targets. A gap penalty of 11 and a gap extension penalty of 1 were both considered standard. To determine the uniqueness of our targets, 83 different species of human microbial flora were compared to the usual gut flora. Based on extensive research, an E-value cut-off of 10^−2^ was selected to differentiate non-homologous proteins [47]. Antibiotic-resistant drug molecules were a primary target since they were unable to interact with human proteins. The non-homologous proteins to the human host and normal intestinal flora proteins were utilized for further assessment.

Further functional characterization of the non-homologous core genome was carried out. Essential genes were considered as those that have a substantial impact on the pathogen’s ability to survive. Therefore, the obtained non-homologous proteins were checked for their essentiality in the Database of Essential Gene (DEG) [35]. A BLAST search with an E-value of 10^−5^ was performed on the non-homolog genes. Moreover, an essential gene database (CEG) was used to look into the genes that had high similarities with DEG. By validating essential genes based on both alignment and function, the CEG reduces the likelihood of receiving false-positive findings from essentiality predictions based on alignment. Overlapping the CEG and DEG essential gene lists allowed us to pick genes with similar reports in both lists for further study.

Using BLAST with an E-value of 10^−5^ against the whole DrugBank dataset [37] of prokaryotes (which include drug targets), the obtained essential genes were analyzed to determine the potential drug targets. For *F. nucleatum*, a list of essential, drug-like proteins with less than 30% identity and more than 50% query coverage against the Drugbank database was identified.

The virulent proteins among the druggable targets were also studied to identify the genes responsible for producing the virulent components using a database called VFDB [48]. Bacterial virulence proteins facilitate bacterial colonization and penetration of host immune cells, hence aiding in the destruction of the host immune system. A threshold of 10^−3^ was used to compare the *F. nucleatum* proteins with the VFDB.

### 2.4. Structural Retrieval and Virtual Screening

A method of in silico structure prediction known as homology modeling may be used to predict the three-dimensional structure of a selected protein, based on its primary amino acid sequence. Drug design requires an understanding of proteins’ 3D structure and function [49]. A selected protein 3-D structure was retrieved from the Alpha Fold server using UniProt sequence ID. We uploaded its sequence to the STRING database online server and found interactions with other proteins, (represented by FN0707).

Virtual screening was performed using lead-like cyanobactins (*n* = 237), streptomycins (*n* = 607), and marine bacterial secondary metabolites (*n* = 1226) against the shortlisted drug target riboflavin kinase. Protonation and energy reduction were used to get the compounds ready for screening. Protein structure, on the other hand, was used as a receptor, while compounds served as ligands. The screening was carried out using MOE version 2019.0102, as described previously [28]. To do the screening, the triangle-matching method was used, whereas London dG, affinity dG, and forcefield parameters were used for rescoring. S-score was used to sort docked molecules. The compound with the lowest binding energy was chosen as the top conformation and used for further investigation.

### 2.5. ADMET Profiling of Shortlisted Drug Candidates

The development of safe drugs is a major problem but the drug development time and expenses may be halved if toxicological side effects can be detected early on. Screened drug-like substances were examined using the SwissADME software [50] for pharmacokinetic properties such as Absorption, Distribution, Metabolism, and Excretion (ADME). pkCSM [51] was used to analyze the compounds’ toxicity profile, minimal human side effects, immunotoxicity, mutagenicity, teratogenicity, neurotoxicity, increased penetration, and carcinogenicity. As a result, the most effective method of administration, the impact on diverse species, and the elimination of drug properties in the human body were evaluated.

### 2.6. Dynamic Simulation Analysis

The complex interaction and stability of the ligand-protein complex were subsequently studied in detail using molecular dynamic (MD) simulation of these molecules. The GROMACS server v2020 [52] was the MD simulation program used, with GROMOS54A71 forcefield to rectify the topologies. The steepest energy minimization algorithm was used to minimize the energy of the ligand topology files. To construct a simulation system, the following parameters were used: size of the box with a 1.0 nm margin from the protein atom border; dodecahedron box as a boundary condition; SPC216 as an accurate water solvation model; Na+ ions were used to neutralize complexes and then simulated for 50 ns at a pressure of 1 atm and a temperature of 300 K. NPT and NVT were considered as an ensemble class, containing 50,000 steps of NPT and NVT [53]. A post-simulation examination of interactions was carried out.

## 3. Results

### 3.1. Pan-Genome Analysis

**Figure 1:**
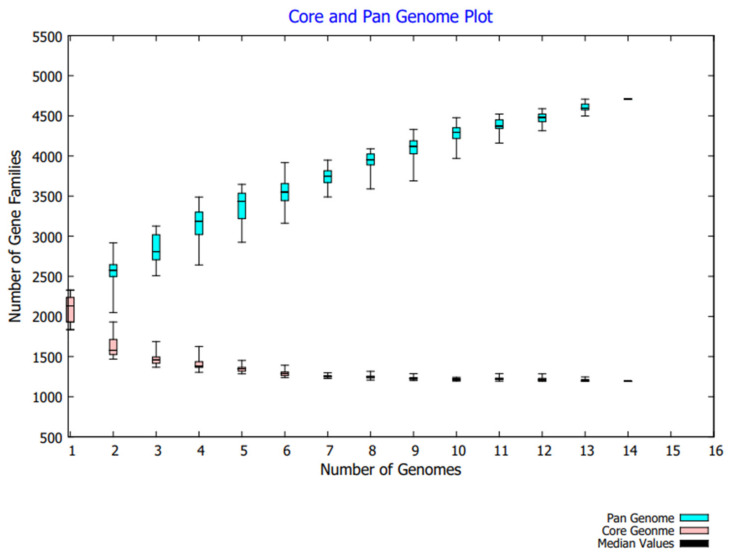
Pan-genome analysis of 14 *F. nucleatum* strains related to CRC.

To prioritize the potent therapeutic target against *F. nucleatum*, the pan-genome analysis was applied to 14 strains. *F. nucleatum* strains consisted of >2230 DNA coding sequences. Among these, 1193 genes were shared by all strains and classified as a core genome fraction (i.e., 56% of pan-genome). The comparative study showed that the *Fusobacterium nucleatum* subsp. animalis strain P2_CP consisted of the maximum number of accessory genes (*n* = 1017), whereas the *F. nucleatum* subsp. polymorphum strain THCT15E1 strain encompassed the maximum number of unique genes (*n* = 211). Only one unique gene was present in the *F. nucleatum* subsp. animalis strain P2_CP while the minimum number of accessory genes were in the strain *F. nucleatum* Fn10-CTX3 (*n* = 567). Additionally, 11 strains were lacking several genes that were present in other strains (File S1). The pan-genome curve represented the B_pan_ = 0.320 (i.e., <1) resulting in the open nature of the pathogen (Figure 1). However, it is hypothesized that the pan-genome will continue to be accessible to new bacterial species as long as evolution and horizontal gene transfers are there [34].

**Figure 2:**
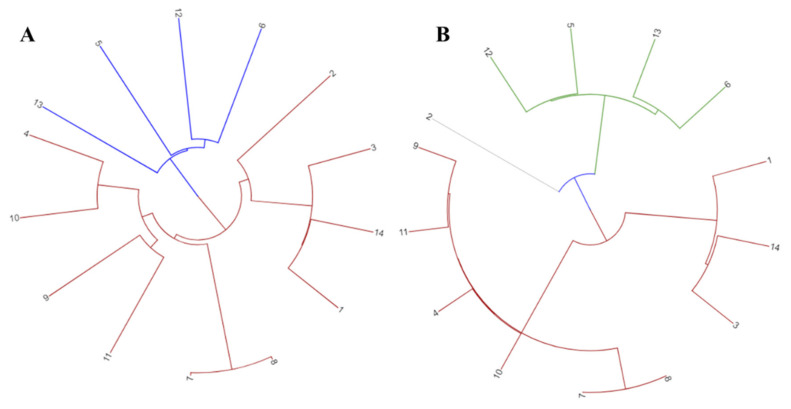
Phylogenetic tree depicting (**A**) Pan-genome phylogeny of 14 CRC related *F. nucleatum* strains used in this study (**B**) Core genome phylogeny of studied *F. nucleatum* strains. 1 = *F. nucleatum* CC53; 2 = *F. nucleatum* Fn10-CTX3; 3 = *F. nucleatum* Fn146CP; 4 = *F. nucleatum* Fn173CP; 5 = *F. nucleatum* Fn3760T; 6 = *F. nucleatum* FnS0431; 7 = *F. nucleatum* subsp. animalis strain P2_CP; 8 = *F. nucleatum* subsp. animalis strain P2_LM; 9 = *F. nucleatum* subsp. animalis strain THCT5A4; 10 = *F. nucleatum* subsp. animalis strain THCT6B3; 11 = *F. nucleatum* subsp. animalis strain THCT7A2; 12 = *F. nucleatum* subsp. polymorphum strain THCT7E2; 13 = *F. nucleatum* subsp. polymorphum strain THCT15E1; 14 = *F. nucleatum* subsp. vincentii strain THCT14A3.

All 14 strains were grouped together into a phylogenetic tree using data from the pan and core genomes. There was a difference in distance between the pan and core genome-based tree branches (Figure 2). Phylogenetic variation was also shown by cluster analysis using both genomic fractions. In comparison with the pan-genome, the distance in the core gene tree branches was much reduced.

### 3.2. Functional Annotation Studies

**Figure 3:**
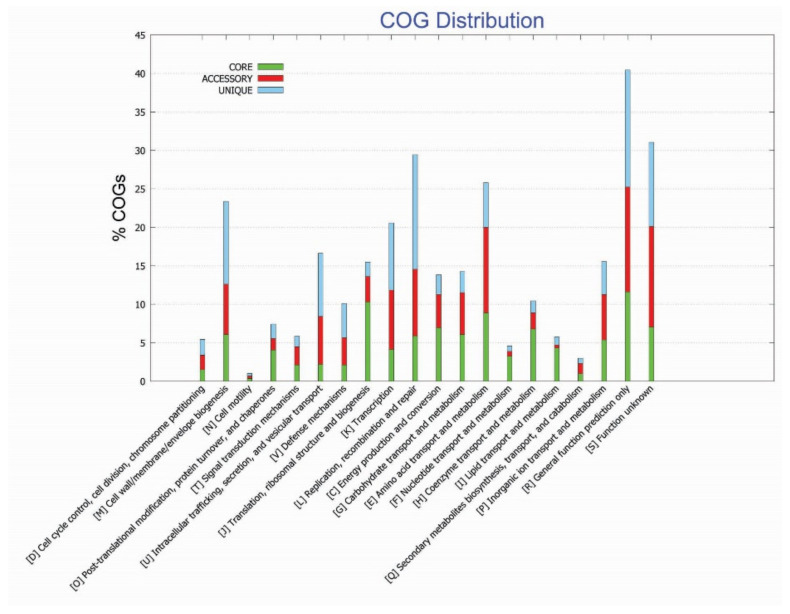
Functional enrichment analysis of identified genome of *F. nucleatum* strains.

The COG functional analysis of the pan-genome was performed to look into the conserved proteins related to their specific metabolic pathways. The COG functional annotation for pan-genome analysis revealed that the core genome was found to be enriched in metabolic pathways, i.e., information storage, processing, and metabolic related pathways mainly enriched in amino acid transport metabolism, ribosomal, translational/biogenesis pathways, and inorganic ions transport metabolism. The accessory genome was identified to be highly involved in information storage, processing, and metabolic related pathways, mainly enriched in amino acid transport metabolism, and having genes linked with general function prediction. However, unique genes were mainly involved in information storage and processing pathways and poorly characterized (functionally unknown) pathways, i.e., general function prediction, cell wall, cell membrane, and envelop biogenesis (Figure 3).

### 3.3. Potential Drug Target Identification

For the purpose of predicting genes necessary for metabolic pathways and the overall survival of the *F. nucleatum*, a differential analysis of 1193 core protein-coding sequences was performed. The non-paralogous proteins from the core genome were retrieved via the CD-HIT tool. It resulted in the identification of 1190 proteins as non-paralogous while three proteins were excluded as paralogous to *F. nucleatum.* These identified 1190 proteins were further used for drug target identification.

Potentially useful and novel therapeutic treatment targets are those that have been highly conserved throughout evolution and are found in almost all pathogen strains of a genus. This explains why the study of such genes has long been a priority for researchers. RNA transcript inhibition and gene knock-out techniques, which are similar to mutant insertion for the loss of function in the gene, are usually used to verify the necessity of genes [54]. In recent decades, it has been possible to integrate this kind of traditional laboratory-based data into an online database, making the process more efficient, uniform, and repeatable. In this study, we evaluated the *F. nucleatum* essential genes using two databases, DEG and CEG. DEG predicts essential genes using a homology-based search algorithm with ~30,000 genes collected from ~70 different species [35], while CEG makes use of a forecasting technique using pre-determined homology-dependent clusters, which allows for the depiction of preservation and the specificity of gene functions. The DEG findings showed that 613 genes were crucial to the pathogen’s survival, whereas the CEG study yielded 550 crucial genes; consequently, the predicted number of essential proteins shared by both sets of genes was 538. These crucial genes were retained for future examination.

To prevent unwanted side effects, we need to ensure that the drug is selectively toxic against *F. nucleatum* but not against human and microbial gut flora. This will prevent the drug from binding to the active sites of the homologous proteins in the host [55]. All 538 of the obtained proteins resultant from the intersection of the DEG and CEG analysis were subjected to BLASTP against the human and microbial gut flora proteome to identify the non-homologous proteins. From a total of 538 proteins, BLAST determined 209 to be dissimilar to the human proteome, whereas 48 were dissimilar to the proteome of the human digestive tract microorganisms.

Furthermore, the druggability of these 48 essential, non-homologous proteins was determined through the BLAST against the DrugBank database. It revealed that only 15 proteins had drug target likeability and can act as potential therapeutics against *F. nucleatum*. Moreover, the virulent factors released from pathogenic proteins are mainly involved in the infliction of infections. Around 12 virulent proteins responsible for the pathogenic condition were identified.

### 3.4. Significant and Novel Drug Target Prediction

It has been reported that cytoplasmic proteins are an excellent therapeutic target and may be readily targeted with drugs [56]. Additionally, ~70 percent FDA approved medications are claimed to target enzymatic proteins because of their substantial participation in numerous pathways. Finally, among 12 candidate therapeutic targets, one protein was identified as an essential, non-homologous, druggable target against *F. nucleatum*, i.e., riboflavin synthase (RiS). Based on their cytoplasmic subcellular distribution, length > 100 amino acids, enzymatic nature, and role in important metabolic pathways, this discovered protein was used for structure-based study.

**Figure 4:**
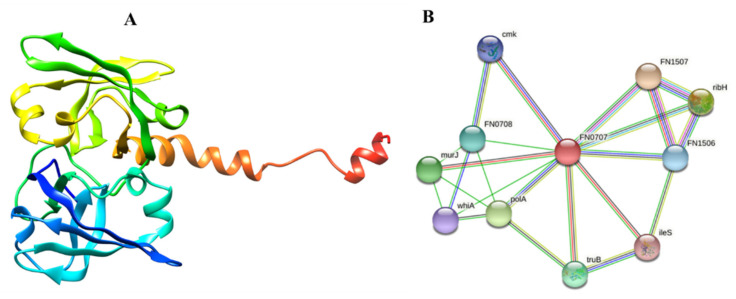
Representation of the selected drug target as a solo protein and part of its network (**A**) 3D structure of RiS retrieved from AlphaFold (**B**) PPI analysis of RiS.

Among these 12 proteins, the enzyme RiS was chosen as the intended target (Figure 4A). Due to its lack of presence in humans, RiS is thought to be a promising therapeutic target since microorganisms are reliant on this metabolic process. The enzyme (EC:2.5.1.9) is essential in the bacterial secondary metabolite biosynthesis (Flavin Biosynthesis I). It catalyzes the final step in the biosynthesis of vitamin B2, i.e., the conversion of two molecules of 6,7-dimethyl-8-ribityllumazine to riboflavin and 5-amino-6-ribitylamino-2,4(1H,3H)-pyrimidinedione.

Tutino et al. [57] show that CRC causes significant changes in the transcription and translation of RFVTs. Additionally, L-methionine and riboflavin metabolisms were the most prominent pathways affected in CRC cells as the cancer advances [58]. Moreover, this is not only in colon cancer as riboflavin is reportedly involved in the onset of gastric cancer [59]. Nevertheless, it is also involved in numerous infectious diseases and is reported as a potential marker for *Candida albicans* [59], *Brucella* spp. [60], and *Aspergillus fumigatus* [61]. While *F. nucleatum* has been linked to colon cancer before, it has never been investigated for therapeutic targets.

Protein-protein interactions and their functional annotation form the backbone of cellular machinery, which is responsible for regulating a plethora of biological activities [62]. To fully comprehend the PPI and its functional significance in the cell, it is necessary to recognize the several interactions that take place and regulate their outcomes [63]. The STRING analysis indicated that our prioritized protein likely interacted with many adjacent proteins to carry out essential functions, suggesting that it is a hub protein. Since proteins often work in groups, inhibiting this one may also impact the activity of its interactors. RiS mediates interactions with other proteins nearby, such as FN1507 (probability score: 0.964), ribH (probability score: 0.960), murJ (probability score: 0.918), FN0708 (probability score: 0.880), FN1506 (probability score: 0.851), cmk (probability score: 0.833), ileS (probability score: 0.819), polA (probability score: 0.935), truB (probability score: 0.891), and whiA (probability score: 0.827). The PPI results showed that isocitrate lyase had a total number of 11 nodes, an average node number of 4.18, an average local clustering coefficient 0.79, a PPI enrichment *p*-value 0.0266, and 15 expected numbers of edges, as shown in (Figure 4B). Many essential processes rely on the presence of these proteins. If RiS is inhibited, the other interactor proteins may eventually become dysfunctional. As a result, RiS may be presented as a viable pharmacological target without apprehension about the repercussions.

### 3.5. Virtual Screening Studies

The 3D structure of RiS was retrieved from the Alphafold server with the identifier ID: AF-Q8RFI9-F1, with 319 amino acids. The retrieved structure was further used for the structure-based docking to prioritize potential inhibitors against it. Molecular docking is an excellent method for determining how studied complexes interact with a biological target, which is crucial in treatment. To comprehend the compounds-RiS interactions and analyze the likely binding mechanism and energy, the produced complexes were studied through the MOE tool. The docking analysis was performed for Ribityl (9-D-ribityl-1,3,7-trihydropurine-2,6,8-trione) as a reference control where the RiS was utilized as a protein receptor. The result showed the binding of the reference compound with the protein with different conformations and orientations. We selected conformation 1 of the ligand based on its binding affinity energy, i.e., −6.40 kcal/mol.

Three natural product libraries were taken and only lead-like compounds were retained for screening, such as (i) cyanobactins (*n* = 237), (ii) streptomycins (*n* = 607), and (iii) marine bacterial secondary metabolites (*n* = 1226), using rigorous docking to the active site of RiS having residues as Gly4, Leu5, Val6, Glu7, Glu8, Lys30, Arg137, Ala143, Ser144, Leu145, Thr146, Val157, Ser158, Leu159, Ile160, His162, Thr163, Lys166, and Ile167. It gave several docked configurations of compounds that were defined by docking scores. Lower binding affinity compounds (i.e., those with a binding affinity less than or comparable to −6 kcal/mol) were excluded from assessment as potential hit candidates. Due to their lower binding affinity than the Ribityl inhibitor, only three compounds (one from each library) were chosen for further study in this work because of their significant inhibitory effect against RiS, i.e., CMNPD3609 (Marine), Malyngamide V (Cyanobactin), and ZINC06804365 (Streptomycin metabolite) to inhibit *F. nucleatum*.

### 3.6. Interaction Analysis of Shortlisted Compounds

**Figure 5:**
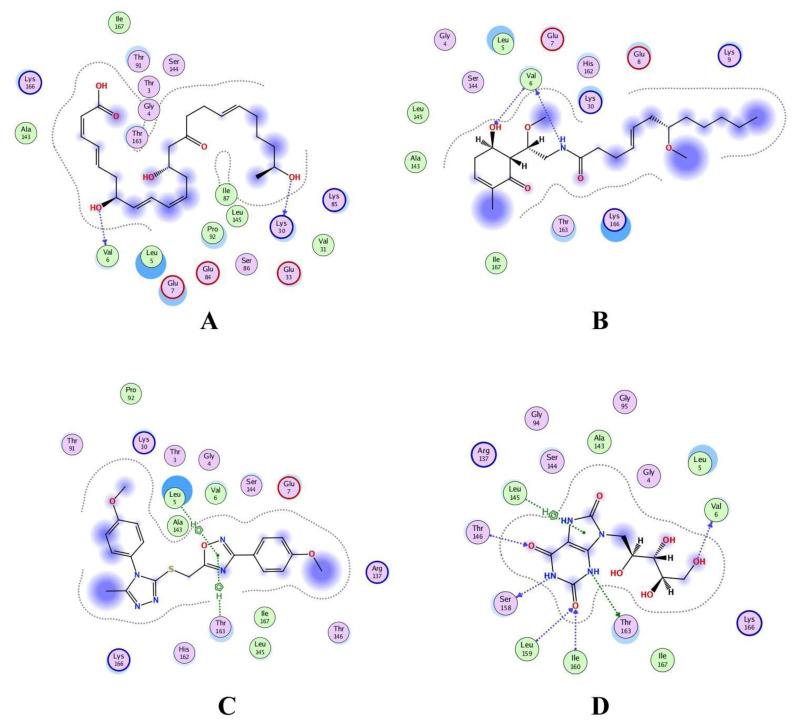
Molecular docking analysis of (**A**) RiS-CMNPD3609, (**B**) RiS-Malyngamide V, (**C**) RiS-ZINC06804365, and (**D**) RiS-control.

Shortlisted compounds were examined using post-molecular docking interaction analysis to better understand RiS pharmacological activity and its binding mechanism. Each ligand had several interactions with the receptor in this molecular docking research. CMNPD3609 (−7.63) > Malyngamide V (−7.03) > ZINC06804365 (−7.01) is the docking rank order based on docking score. The hydroxyl group of the CMNPD3609 site chain mediates two hydrogen bonds with Lys30, and Val6, each with a bond distance of 3.18 Å and 2.94 Å along with an energy of −0.8 and −1.8 kcal/mol, correspondingly (Figure 5A). Malyngamide V, through its aromatic ring-OH, mediates two hydrogens bind with Val6 having a bond distance of 3.07 Å and an energy of −1.2–2.8 kcal/mol (Figure 5B). ZINC06804365 showed two pi-hydrogen interactions between Leu5, Thr163, and the aromatic ring mediates, with a bond distance of 4.28–4.44 Å and an energy range of −1.6 to −0.5 kcal/mol (Figure 5C). The control compound docked with RiS at −6.40 kcal/mol formed six hydrogen bonds with Ser158, Thr163, Val6, Leu159, Ile160, and Thr146 whereas its aromatic ring mediates a single pi-hydrogen bond with the Leu145 residue (Figure 5D). A description of binding interactions formed inside the RiS active cavity by the selected compounds is shown in Table 2.

### 3.7. ADMET Profiling of Shortlisted Drug Candidates

To avoid drug adverse reactions and toxicity, the computational evaluation of ADMET properties is one of the crucial steps to investigate the drug safety assessment at the initial step of a drug candidate. The shortlisted four compounds (three natural products and one standard) were found to be non-inhibitors of CYP2C19 and CYP2D6, with significant permeability to skin and Caco2. Only CMNPD3609 was identified as a non-inhibitor while the other two compounds were identified as P-glycoprotein inhibitors, whereas all three prioritized compounds were impermeable to the BBB.

The ADMET characterization is a significant part of the drug discovery process since it reduces costs and development times in clinical trials [64]. When categorizing compounds for analogies to drugs, the Lipinski rule of five was used as a preliminary step. Ideally, drug-like molecules would have lipophilicity values (LogP) below five, hydrogen bond acceptor sites below ten, and a molecular weight of 500 amu or less. This is the ideal molecular weight range for orally administered drugs and chemicals, as suggested by RO5. This resulted in the finding that all three compounds precisely adhered to the RO5 guidelines as shown in Table 3.

Additionally, the Ames mutagenesis study used the pkcsm tool to identify the chemicals and predict their toxicity, Max. tolerated dose (human), Minnow toxicity, *T. Pyriformis* toxicity, Oral Rat Acute Toxicity (LD50), Hepatotoxic, and Skin Sensitization evaluation. Consequently, all compounds showed a negative Ames test except CMNPD3609. This means that these molecules do not cause mutagenicity. Only ZINC6804365 showed hepatoxicity while all compounds were observed to have no skin sensitization. *T. pyriformis* showed the maximum tolerance to Malyngamide-V (0.575 log ug/L), while less tolerance was seen for the remaining compounds. The detailed information is shown in Table 3.

### 3.8. MD Simulation of Protein-Ligand Complex

**Figure 6:**
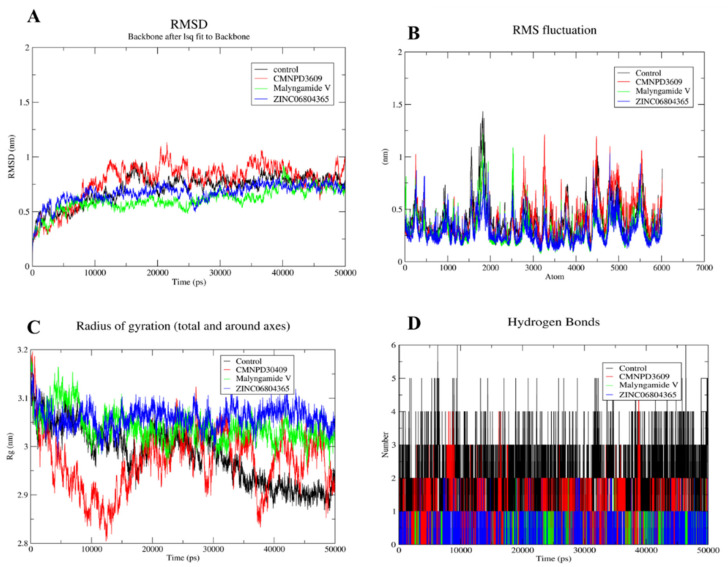
MD simulation for shortlisted compounds, depicting (**A**) RMSD (**B**) RMSF (**C**) Radius of gyration and (**D**) Hydrogen bonds.

The MD simulation was performed for the shortlisted inhibitors to validate the complex interactions and flexibility. The GROMACS server was used to find the movements of molecules and atoms of protein complexes at 50 ns. All the compounds showed mild to moderate fluctuations within the range of 1.5–2 Å while all compounds were observed to be stable after 5 ns (Figure 6A). The RMSF and Rg for all four compounds highlights the identical patterns of protein and its binding pocket stability (Figure 6B,C). Importantly, the binding pattern of these compounds showed that the control mediated five hydrogen bonds, CMNPD3609 made four hydrogen bonds, while Malymgamide V and ZINC06804365 mediated three to four hydrogen bonds throughout the 50 ns simulations (Figure 6D). CMNPD3609 was observed to have high to moderate fluctuation in terms of RMSD, and a radius of gyration, while Malymgamide V and ZINC06804365 formed a stable complex throughout the simulation.

## 4. Discussion

CRC is a pressing health issue worldwide [65], with a proportion of 9.2% among various cancer diagnoses and the second highest fatality rate of any cancer type [66]. The underlying mechanism of CRC malignancy is still to be determined but inflammation has been recognized as an essential risk factor for colorectal cancer, which is produced by a complex interaction of environmental, dietary, behavioral, and hereditary variables. The anaerobic, gram-negative bacillus *F. nucleatum* lives in species-specific reservoirs throughout the human body, including the mouth, gastrointestinal system, and other sites. Analysis of the 16S ribosomal ribonucleic acid (rRNA) gene sequence and the use of metagenomic sequencing techniques revealed a strong association between *F. nucleatum* and CRC [67]. Compared with the control group, it was significantly higher in patients with CRC [68]. Additionally, *F. nucleatum* was associated with a worse prognosis for CRC patients and likely aided in the development of chemoresistance [69].

Since pan-genomics has evolved as a standard for understanding the molecular evolution of bacterial populations in respect to the ever-increasing diversity of bacterial genomes [70], we analyzed pan-genomes to study species specific variations in *F. nucleatum*. The analysis of the 14 *F. nucleatum* genomes isolated from the CRC patients revealed the presence of various numbers of unique genes and accessory genes, with a shared core genome amounting to 1193 genes. Among the identified 12 potential therapeutic markers of *F. nucleatum* related to CRC, the RiS was selected as a novel and promiscuous drug/therapeutic prognostic target/marker involved in the biosynthesis of the secondary metabolite Vitamin B12. Its reported role in CRC makes it a highly relatable therapeutic marker and drug target for CRC control. Tutino et al. [57] have shown that CRC causes significant changes in the transcription and translation of riboflavin transporters [57]. Additionally, L-methionine and riboflavin metabolisms were the most prominent pathways affected in CRC cells as the cancer advances [58]. Riboflavin synthase has also been previously earmarked as a drug target in several pathogenic bacteria [71,72,73], with an effect on the host’s immune response. Since *F. nucleatum* is generally linked with immune suppression in CRC, inhibiting it with RiS binding of drugs can escalate the host cell pathogen clearance. Therefore, RiS inhibition can be a two-pronged approach, where both pathogen and host immunity can be targeted through simultaneous bactericidal activity and mitigation of host cell inflammation. Therefore, virtual screenings of lead-like natural products from various sources were performed to prioritize a new potent drug candidate against RiS, using Ribityl (9-D-ribityl-1,3,7-trihydropurine-2,6,8-trione) as a reference control. It resulted in the shortlisting of three drug candidates as potential binders, i.e., CMNPD3609 (Marine), Malyngamide V (Cyanobactin), and ZINC06804365 (Streptomycin metabolite) to inhibit *F. nucleatum*. The ZINC06804365 has been tested and reported as a potential inhibit for many signaling pathways such as Wnt-3a and also as having inhibitory activity against the Tankyrase-2 enzyme which reportedly has anti-cancer activities [74]. Malyngamide V has been widely studied for its anti-inflammatory and antinociceptive role [75] and CMNPD3609 possesses several antibacterial activities [76]. The diverse role of these compounds in different cancer and antimicrobial activities makes them a possible drug candidate to curb CRC caused by *F. nucleatum*. However, the discovery of these compounds as drug candidates for CRC or *F. nucleatum* infections needs further experimental studies to validate their function. Furthermore, MD simulation was performed for these shortlisted compounds for 50 ns, to identify whether the binding and inhibition would be long lasting or transitory. It showed stable binding of all complexes (fluctuation within the range of 1.5–2 Å) after the initial phase of ~5 ns.

Eventually, the current computational pipeline will help in the identification of natural therapeutic products against the targets prioritized by the high throughput screening of the *F. nucleatum* core genome. Assuming the hefty cost, coupled with the small success rate in drug discovery and development, repurposing available compounds or the screening of natural product libraries against new targets and diseases is an important venture. It has lower development budgets and shorter screening periods, saving cost and time. Since natural products have been used in traditional medicine for hundreds of years, they are ideal for drug repositioning due to their medicinal value. However, this approach is best for prioritization although limitations exist. Therefore, we suggest extensive lab analysis in model organisms followed by clinical trials.

## 5. Conclusions

Cancer rate is increasing and complications associated with it, including role of microbes is a complex science. We need to maneuver the intricacies of the diseases like CRC, through swift approaches that are based on computation and artificial intelligence, both by understanding the mechanisms and finding cure for it. Bioinformatics based subtractive genomic approach is a state of the art method for identifying drug targets from genome sequences. Herein, we utilized this approach for finding out the therapeutic proteins from the CRC related oncomicrobe *F. nucleatum* genomes. MOE was then used for screening out inhibitors from natural product libraries against the selected target riboflavin synthase. Although, we got quick results but other parallel approaches may also be used for screening inhibitors and compare to our findings. The procedure could be further improved to Kdi or MIC evaluation of these inhibitors using artificial intelligence algorithms. Shortlisted natural product inhibitors may further be tested in the laboratory.

## Figures and Tables

**Table 1:** *F. nucleatum* genomes used in this study, either derived from colon cancer site, tissue, or gut of the ailing person.

Serial No.	Name	Biosample Accession	Genome Size (Mbp)	Isolation Source	Coding DNA Sequences	Reference
1.	*F. nucleatum* Fn146CP	SAMN20819806	2.082	Tissue	1949	[38]
2.	*F. nucleatum* Fn10-CTX3	SAMN20819805	2.101	Tissue	2026	[38]
3.	*F. nucleatum* Fn3760T	SAMN20819808	2.299	Tissue	2234	[38]
4.	*F. nucleatum* Fn173CP	SAMN20819807	2.121	Tissue	2041	[38]
5.	*F. nucleatum* FnS043-1	SAMN20819807	2.288	Tissue	2245	[38]
6.	*F. nucleatum* subsp. animalis strain THCT5A4	SAMN20819807	2.491	Gut	2360	[39]
7.	*F. nucleatum* subsp. polymorphum strain THCT15E1	SAMN18042967	2.526	Gut	2405	[39]
8.	*F. nucleatum* subsp. animalis strain THCT7A2	SAMN18042965	2.515	Gut	2339	[39,40]
9.	*F. nucleatum* subsp. polymorphum strain THCT7E2	SAMN18042966	2.547	Gut	2420	[39]
10.	*F. nucleatum* subsp. vincentii strain THCT14A3	SAMN18042968	2.053	Gut	1903	[39]
11.	*F. nucleatum* subsp. animalis strain THCT6B3	SAMN18042964	2.269	Gut	2116	[39]
12.	*F. nucleatum* subsp. animalis strain P2_CP	SAMN07448031	2.351	Colorectal primary tumor	2346	[41]
13.	*F. nucleatum* subsp. animalis strain P2_LM	SAMN07448032	2.346	Liver metastasis	2353	-
14.	*F. nucleatum* CC53	SAMN02469329	2.070	Colon adenocarcinoma	1879	[42]

**Table 2:** Docking scores and identified bond types predicted through MOE tool for shortlisted compounds within the binding cavity of RiS.

S. No.	Ligand	Receptor	Interaction	Distance	E (kcal/mol)	S Value (kcal/mol)
1	Ribityl control					−6.40
	N 2	O SER 158	H-donor	2.87	−5.9	
	N 4	OG1 THR 163	H-donor	3.06	−2.0	
	O 21	O VAL 6	H-donor	2.91	−1.4	
	O 9	CA LEU 159	H-acceptor	3.43	−0.5	
	O 9	N ILE 160	H-acceptor	3.22	−2.8	
	O 10	N THR 146	H-acceptor	2.95	−2.5	
	5-ring	CA LEU 145	pi-H	4.49	−1.0	
2	CMNPD3609					−7.63
	O 27	O LYS 30	H-donor	3.18	−0.8	
	O 56	O VAL 6	H-donor	2.94	−1.8	
3	Malyngamide V					−7.03
	N 13	O VAL 6	H-donor	3.08	−2.8	
	O 7	N VAL 6	H-acceptor	3.07	−1.2	
4	ZINC06804365					−7.01
	5-ring	CA LEU 5	pi-H	4.44	−1.6	
	5-ring	CG2 THR 163	pi-H	4.28	−0.5	

**Table 3:** Three shortlisted compounds as possible drug candidates against RiS along with their ADMET properties.

**Compounds ID**	CMNPD3609	Malyngamide-V	ZINC6804365
**Compound Name**	Isomacrolactic Acid	Malyngamide-V	34M (PDB ID)
**Structure**	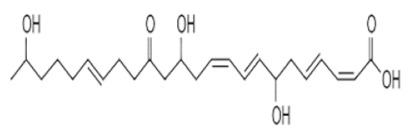	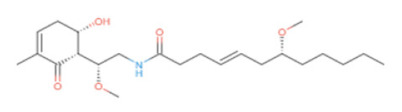	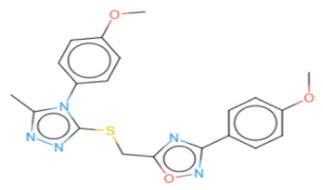
**GI absorption**	100	92.819	98.36
**Caco2**	1.317	1.076	1.242
**Water solubility**	−0.102	−4.386	−3.651
**Skin permeability**	−2.282	−2.827	−2.735
**BBB permeant**	No		
**Lipinski**	Yes		
**Binding Affinity (kcal/mol)**	−7.63	−7.03	−5.9
**Radar**	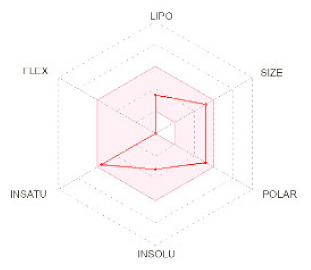	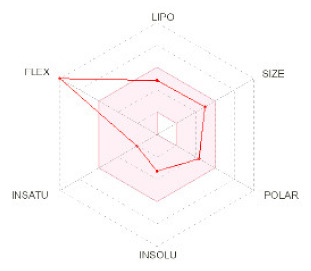	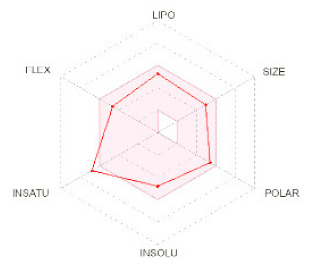
**Ames**	Yes	No	No
**Max. tolerated dose (human)**	0.959	0.21	0.805
**Hepatotoxicity**	No	No	Yes
**Skin sensation**	No	No	No
***T.*** ***Pyriformis* toxicity**	−0.964	0.575	0.286
**Minnow toxicity**	4.806	0.929	−2.353

## Data Availability

All the genomic data used in this manuscript is publicly available from databases like NCBI and BV-BRC server, with accession numbers of genomes mentioned at appropriate places in the manuscript.

## Supplementary Materials

The following supporting information can be downloaded at: https://www.mdpi.com/article/10.3390/cancers14246260/s1, File S1.
